# Differences in gene expression profiles in early and late stage rhodesiense HAT individuals in Malawi

**DOI:** 10.1371/journal.pntd.0011803

**Published:** 2023-12-06

**Authors:** Peter Nambala, Julius Mulindwa, Harry Noyes, Vincent Pius Alibu, Barbara Nerima, Joyce Namulondo, Oscar Nyangiri, Enock Matovu, Annette MacLeod, Janelisa Musaya, on behalf of the TrypanoGEN+ Research Group as Members of the H3Africa Consortium

**Affiliations:** 1 Department of Biochemistry and Sports Sciences, College of Natural Sciences, Makerere University, Kampala, Uganda; 2 Kamuzu University of Health Sciences, Malawi-Liverpool-Wellcome Trust Clinical Research Programme, Blantyre, Malawi; 3 Centre for Genomic Research, University of Liverpool, Liverpool, United Kingdom; 4 Department of Biotechnical and Diagnostic Sciences, College of Veterinary Medicine Animal Resources and Biosecurity, Makerere University, Kampala, Uganda; 5 Wellcome Centre for Integrative Parasitology, University of Glasgow, Glasgow, United Kingdom; University of Antwerp Drie Eiken Campus: Universiteit Antwerpen Campus Drie Eiken, BELGIUM

## Abstract

*T*. *b*. *rhodesiense* is the causative agent of Rhodesian human African trypanosomiasis (r-HAT) in Malawi. Clinical presentation of r-HAT in Malawi varies between foci and differs from East African HAT clinical phenotypes. The purpose of this study was to gain more insights into the transcriptomic profiles of patients with early stage 1 and late stage 2 HAT disease in Malawi. Whole blood from individuals infected with *T*. *b*. *rhodesiense* was used for RNA-Seq. Control samples were from healthy trypanosome negative individuals matched on sex, age range, and disease foci. Illumina sequence FASTQ reads were aligned to the GRCh38 release 84 human genome sequence using HiSat2 and differential analysis was done in R Studio using the DESeq2 package. XGR, ExpressAnalyst and InnateDB algorithms were used for functional annotation and gene enrichment analysis of significant differentially expressed genes. RNA-seq was done on 23 r-HAT case samples and 28 healthy controls with 7 controls excluded for downstream analysis as outliers. A total of 4519 genes were significant differentially expressed (p adjusted <0.05) in individuals with early stage 1 r-HAT disease (n = 12) and 1824 genes in individuals with late stage 2 r-HAT disease (n = 11) compared to controls. Enrichment of innate immune response genes through neutrophil activation was identified in individuals with both early and late stages of the disease. Additionally, lipid metabolism genes were enriched in late stage 2 disease. We further identified uniquely upregulated genes (log2 Fold Change 1.4–2.0) in stage 1 (ZNF354C) and stage 2 (TCN1 and MAGI3) blood. Our data add to the current understanding of the human transcriptome profiles during *T*. *b*. *rhodesiense* infection. We further identified biological pathways and transcripts enriched than were enriched during stage 1 and stage 2 r-HAT. Lastly, we have identified transcripts which should be explored in future research whether they have potential of being used in combination with other markers for staging or r-HAT.

## Author summary

Clinical outcomes of Rhodesian sleeping sickness in endemic countries varies depending on disease foci. To gain more insight of r-HAT disease in Malawi, we analyzed gene expression profiles in blood from 23 individuals with early stage 1 and late stage 2 sleeping sickness versus 21 healthy controls. We found differences in gene expression profiles in individuals with stage 1 and stage 2 disease but not between Malawi’s r-HAT foci. Furthermore, innate immune response transcripts were elevated in individuals with stage 1 sleeping sickness, and there was enrichment of proliferating neutrophil precursors in cases versus controls. We have also shown that wasting in individuals with late stage sleeping sickness might be linked with altered lipid metabolic process. Lastly, we have identified transcripts that in combination with other markers maybe explored in future for possibility of being used in staging of sleeping sickness without the need of the invasive lumber puncture. Our findings add to the current understanding of human response to *T*. *b*. *rhodesiense* infection in Malawian r-HAT patients.

## Introduction

Rhodesiense human African trypanosomiasis (r-HAT) or sleeping sickness impacts on health and economic burdens in resource limited areas of sub-Saharan Africa. r-HAT is caused by *Trypanosoma brucei rhodesiense* (*T*. *b*. *rhodesiense*) and transmitted by tsetse flies of the genus Glossina [1]. Sleeping sickness is characterized by a hemolymphatic stage 1 (early) and meningoencephalitic stage 2 (late) disease, and is fatal if treatment is delayed [2]. Accurate diagnosis of r-HAT is key in reducing disease burden and mortality [3]. Unfortunately, r-HAT diagnosis in endemic areas is dependent on insensitive microscopic examination of blood and the invasive cerebral spinal fluid (CSF) collection for accurate staging prior to treatment commencement due to toxicity of HAT drugs [4,5].

The clinical outcomes of sleeping sickness vary in endemic countries [6]. For instance, r-HAT cases in Uganda frequently present with an acute clinical disease in contrast to a chronic disease in Malawi [7]. Most cases in Malawi’s Nkhotakota focus present with a chronic stage 1 disease compared to an acute stage 2 disease in Rumphi focus [8,9]. Variations in clinical presentation of r-HAT has been associated with *T*. *b*. *rhodesiense* genetic diversity and the human inflammatory cytokine responses [6,10].

Transcriptome analysis of peripheral blood in Ugandan r-HAT patients identified functional enrichment of genes involved in innate immune response pathway during stage 1 of the disease [11]. The genes included interleukin 21 *(IL21)*, interleukin 1 receptor *(IL1R)*, tumour necrosis factor alpha *TNFA*, immunoglobulin heavy chain variable and classical complement pathway genes [11]. Whereas, upregulated transcripts in the CSF of stage 2 HAT patients were predominantly coding for genes involved in neuro activation and anti-inflammatory pathways. The study also identified *IGHD3–10*, *C1QC* and *MARCO* genes as having a fivefold change in stage 1 r-HAT cases compared to healthy controls [11]. It remains unknown whether or not the transcriptome profiles of r-HAT patients in other endemic countries is the same. The aim of this study was first to determine differential gene expression profiles of stage 1 and stage 2 r-HAT cases with respect to uninfected controls in Malawi and secondly compare transcriptome profiles in r-HAT cases between Nkhotakota and Rumphi foci. Our results add to the current understanding of the human response to r-HAT disease and have led to the identification of potential blood markers that should be validated in future research if they may be used for staging of r-HAT within Malawi.

## Results

### RNA-Seq sample attributes

Samples were collected at Rumphi and Nkhotakota district hospitals during HAT surveillance as previously described [8]. In Rumphi district, a total of 37 r-HAT positive cases and 25 corresponding r-HAT negative controls were recruited **(S1 Table)**, of which 26/37 (70.3%) individuals were males and 11/37 (29.7%) were females. The mean age of the cases and controls were 34.9±17.2 years and 36.0±17.7 years, respectively.

In Nkhotakota district, 27 r-HAT cases were recruited together with 24 corresponding negative controls **(S1 Table)**. Among the cases, 15/27 (55.6%) were males and 12/27 (44.4%) females. The mean age of the cases and controls were 27.2±17.7 years and 33.1±11.8 years respectively.

After RNA quality control, RNA-Seq data was obtained from 23 r-HAT cases and 28 healthy control blood samples that had total RNA concentration ≥ 1μg from both Rumphi and Nkhotakota districts **(Table 1).**

**Table 1:** Summary of blood samples from r-HAT cases and healthy controls used for RNA sequencing.

*Sample ID*	*Phenotype*	*HAT Foci*	*Sex*	*Age*	*r-HAT Stage*	*RNA (μg)*
*MN03TR*	Case	Nkhotakota	F	18	1	1.1
*MN04TR*	Case	Nkhotakota	F	21	1	1.58
*MN06TR*	Case	Nkhotakota	M	25	1	1.62
*MN07TR*	Case	Nkhotakota	M	56	1	1.18
*MN09TR*	Case	Nkhotakota	M	24	2	>2.0
*MN010TR*	Case	Nkhotakota	F	10	1	1.54
*MN011TR*	Case	Nkhotakota	F	21	2	>2.0
*MN012TR*	Case	Nkhotakota	M	24	1	>2.0
*MN013TR*	Case	Nkhotakota	F	6	1	>2.0
*MN014TR*	Case	Nkhotakota	M	4	1	>2.0
*MN019TR*	Case	Nkhotakota	M	56	1	>2.0
*MN020TR*	Case	Nkhotakota	F	40	1	1.08
*MN024T*	Case	Nkhotakota	M	28	2	8.8
*MN030T*	Case	Nkhotakota	F	15	2	2.56
*MN031T*	Case	Nkhotakota	M	17	1	2.56
*MN034T*	Case	Nkhotakota	M	22	2	9.72
*MR039TR*	Case	Rumphi	M	46	2	>2.0
*MR041TR*	Case	Rumphi	M	26	2	1.72
*MR044TR*	Case	Rumphi	M	32	1	1.64
*MR102TR*	Case	Rumphi	M	24	2	3.04
*MR036TR*	Case	Rumphi	M	60	2	>2.0
*MR105TR*	Case	Rumphi	F	28	2	4.43
*MR106TR*	Case	Rumphi	M	30	2	3.8
*MR016CR*	Control	Rumphi	M	48	N/A	1.42
MR021CR*	Control	Rumphi	F	16	N/A	1.04
MR022CR*	Control	Rumphi	F	45	N/A	1.14
*MR025CR*	Control	Rumphi	M	10	N/A	1.95
*MR037CR*	Control	Rumphi	M	69	N/A	0.91
*MR102CR*	Control	Rumphi	M	36	N/A	3.12
MR104CR*	Control	Rumphi	M	36	N/A	1.95
*MR105CR*	Control	Rumphi	M	35	N/A	2.32
MR106CR*	Control	Rumphi	M	65	N/A	3.84
*MN03CR*	Control	Nkhotakota	F	28	N/A	4.72
*MN04CR*	Control	Nkhotakota	F	29	N/A	12.36
*MN06CR*	Control	Nkhotakota	M	30	N/A	4.04
MN08CR*	Control	Nkhotakota	F	20	N/A	3.50
*MN09CR*	Control	Nkhotakota	M	24	N/A	>2.0
*MN010CR*	Control	Nkhotakota	F	27	N/A	4.64
*MN011CR*	Control	Nkhotakota	F	25	N/A	5.60
*MN012CR*	Control	Nkhotakota	M	23	N/A	2.68
*MN013CR*	Control	Nkhotakota	F	27	N/A	2.78
*MN014CR*	Control	Nkhotakota	M	30	N/A	4.36
*MN019CR*	Control	Nkhotakota	M	54	N/A	3.46
*MN020CR*	Control	Nkhotakota	F	30	N/A	3.24
*MN024CR*	Control	Nkhotakota	M	28	N/A	1.86
MN026CR*	Control	Nkhotakota	M	32	N/A	6.36
*MN027CR*	Control	Nkhotakota	M	46	N/A	5.08
MN028CR*	Control	Nkhotakota	M	68	N/A	2.36
*MN030CR*	Control	Nkhotakota	F	31	N/A	3.26
*MN031CR*	Control	Nkhotakota	M	23	N/A	2.30
*MN034CR*	Control	Nkhotakota	M	23	N/A	4.48

*Control samples that were outliers and excluded for all downstream RNA-seq analysis.

### Transcriptome profiles in r-HAT cases and control are different

**Fig 1:**
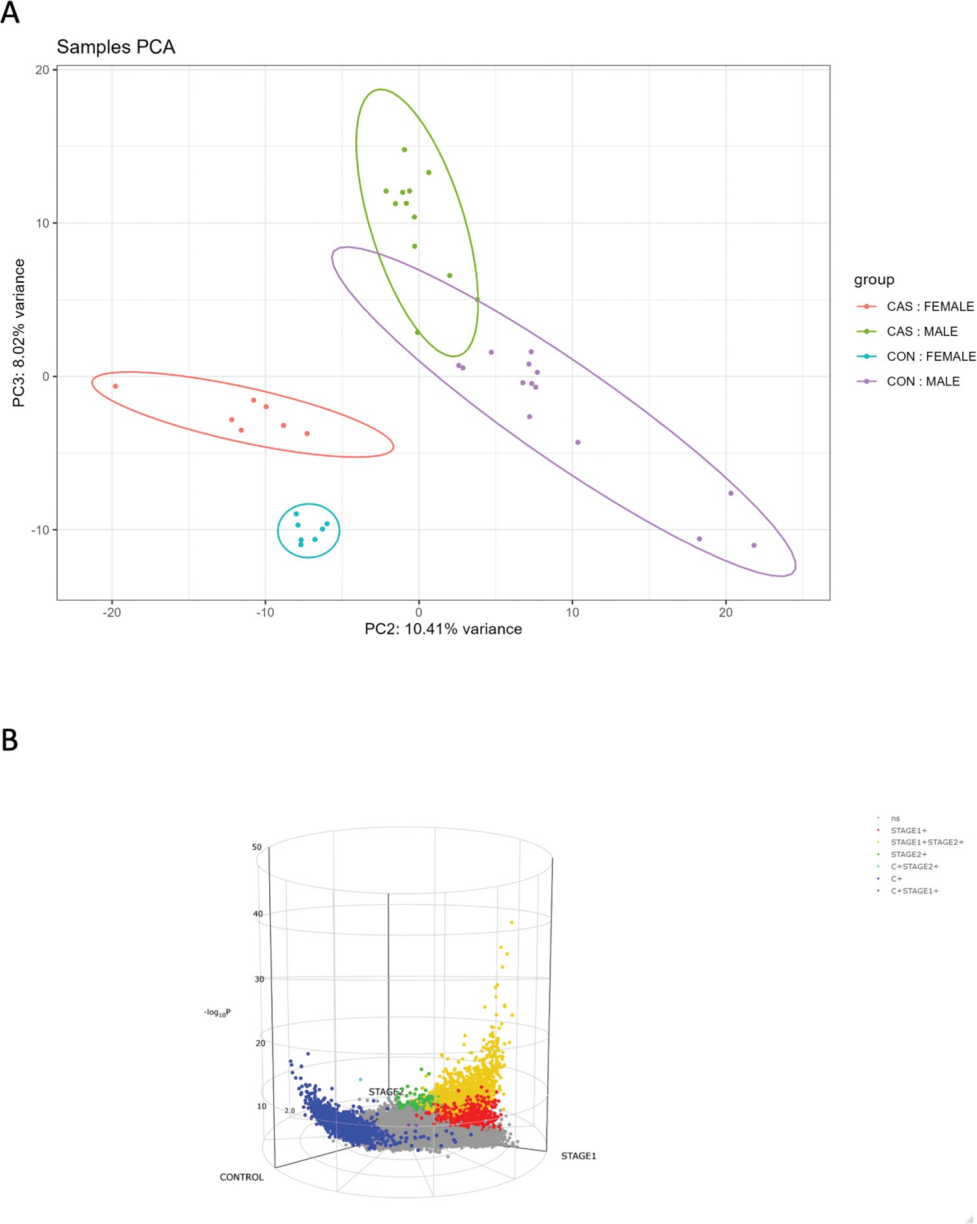
Stratification of Differentially Expressed genes (DEGs) in cases versus controls. **(A)** Principal component analysis (PCA) values for r-HAT cases vs healthy controls grouped into males and females which were compared on Principal Component (PC) 3 and PC2. **(B)** 3D volcano plot showing distribution and relationship of DEGs in Stage 1, Stage 2 and Controls. Grey dots represent non-significant genes, dark blue dots are genes expressed in controls only, red dots are genes expressed in stage 1 only, green dots are genes expressed in stage 2 only, orange are genes expressed in both stage 1and stage 2, purple dots are genes expressed in controls plus stage 1 and light blue dots are genes expressed in controls plus stage 2.

To examine the differences between blood transcriptomes of individuals infected with *T*. *b*. *rhodesiense* compared with healthy controls, we performed a principal component analysis (PCA) in DESeq2 [12]. Seven control samples were outside the 95% confidence ellipse in PCA and excluded in PCA comparison of cases versus controls. The results showed that transcriptomes in male and female individuals infected with *T*. *b*. *rhodesiense* were clearly distinguished from healthy controls on a plot of principal components (PC) 1 and 2 (**Fig 1A**). We also observed a stratification with the same comparison using Euclidean distance correlation (**S1A Fig**). Furthermore, we observed significant differentially expressed genes (DEGs) between stage 1 and stage 2 samples against controls **(Figs 1B and S1B)**. Since clinical presentation of r-HAT in Malawi is foci dependent [8], next we compared transcriptome profiles of cases between Nkhotakota and Rumphi foci. However, there were no genes that were significant differentially expressed between cases in the two r-HAT foci.

### Innate immune response transcripts are elevated in Stage 1 patients

**Fig 2:**
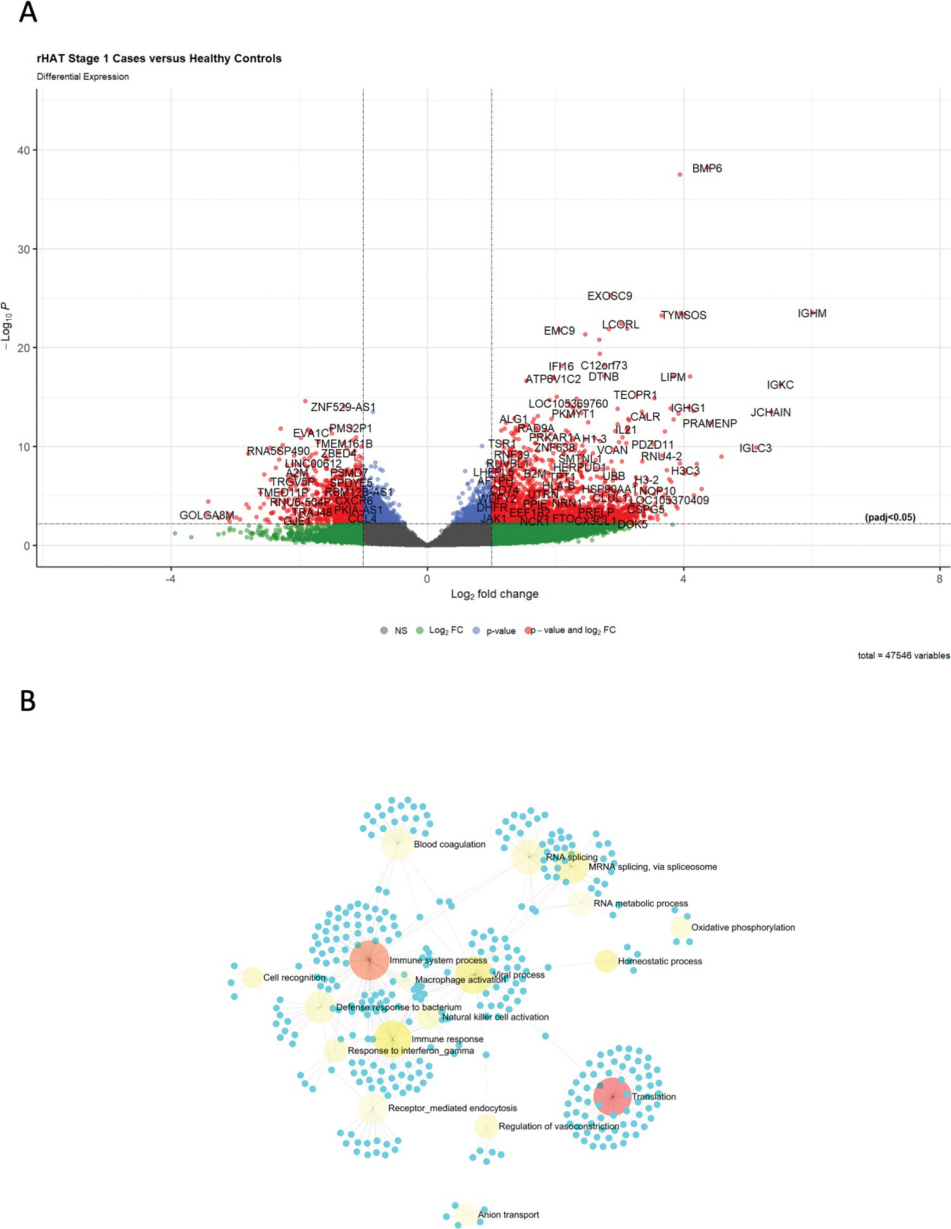
DEGs and network analysis in Stage 1 case blood. **(A)** Volcano plot showing genes that were significant (padj< 0.05) DEG, upregulated (log2FC > 1.0) and downregulated (log2FC < -1). **(B)** ExpressAnalyst network graph of upregulated protein coding genes. The root of the nodes was color coded according to significance with light yellow representing less significant and red more significant. Translation and immune system process were the most enriched biological pathways in stage 2 blood.

Given the differences observed in the number of DEGs between r-HAT stage 1 and stage 2 blood relative to controls, next we sought to identify genes that are significantly enriched in individuals with stage 1 r-HAT disease. First, differential transcriptome analysis was done in stage 1 cases against healthy controls using DESeq2. We also checked whether the blood transcriptome profiles in children aged <10 years were different from those of adults, we found that the children clustered within the 95% confidence ellipse of the adults (**S2 Fig**). In total, 4519/47546 (9.50%) genes were significant (adjusted p, padj<0.05) differentially expressed in stage 1 cases of which 54.3% (2454/4519) were proteins coding genes (**Figs 2A, and S3A, and S1 File**). Among the protein coding genes, 64.6% (1585/2454) were upregulated (log2 fold change, log2FC > 1). Upregulation of immunoglobulin light chains (IGKs, IGLs), immunoglobulin heavy chains (log2FC 2.0–6.0) and Interleukin (IL)-21 (*IL21*) (log2FC 3.1) were also observed. In addition, we observed that *clock interacting pacemaker* (*CIPC)* was differentially expressed (padj<1.59E-6) and down regulated (log2FC -1.9) in peripheral blood. However, *period circadian regulator 1* (*PER1*) transcripts were not significant (padj<0.05) differentially expressed but were dysregulated (log2FC 1.7).

Upregulated genes were uploaded in ExpressAnalyst using the PANTHER biological process database [13,14], and identified 18 biological processes that were enriched in stage 1 cases **(Fig 2B)**. Functional annotation of the principal component gene ontology [15], also identified immune system function as having the most enriched genes with high loadings on the selected principal components **(S2 Table)**.

### Enrichment of lipid metabolic process pathway in stage 2 r-HAT cases

**Fig 3:**
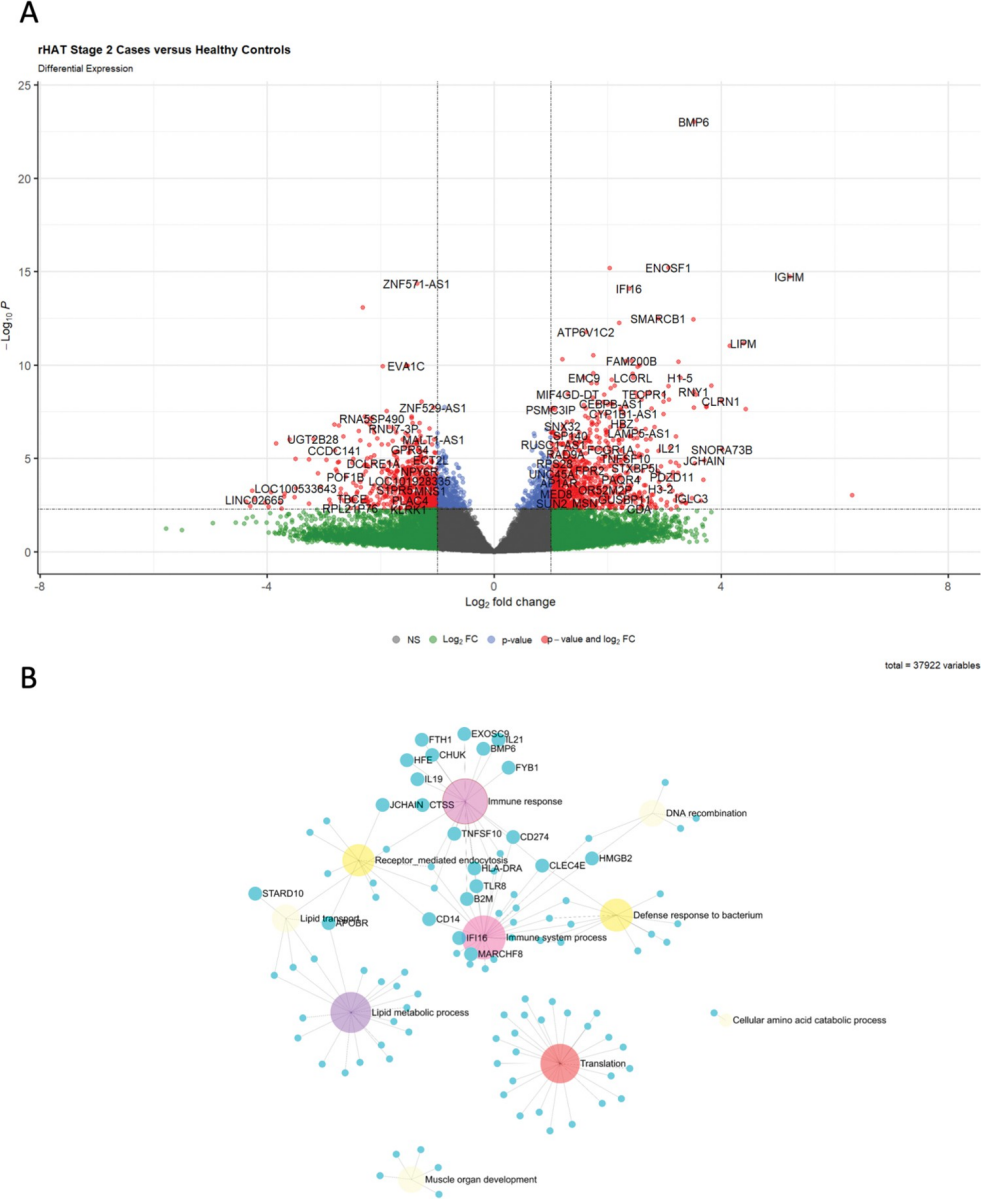
DEGs and network analysis in blood from Stage 2 case versus healthy controls. **(A)** Volcano plot showing **s**ignificant DEGs (padj< 0.05) that were upregulated (log2FC > 1.0) and downregulated (log2FC < -1.0). **(B)** ExpressAnalyst network graph of protein coding genes that were upregulated in stage 2 blood relative to controls. The root of the nodes was color coded according to significance with light yellow representing less significant and red more significant. Translation, immune system process and lipid metabolic process were the most enriched biological pathways in stage 2 blood.

To determine blood transcriptomes that were enriched in stage 2 patients, we compared stage 2 transcriptomes against heathy controls and there were 1824/37922 (4.81%) significant DEGs (padj<0.05) **(Figs 3A and S3B, and S2 File)**. Upregulated protein coding genes (375/850) were analysed in ExpressAnalyst to identify biological process pathways enriched in the PANTHER biological process database. This identified translation (padj<9.19E-6), immune system process (padj<3.59E-4) and immune response (padj<0.004) as the most significant enriched biological pathways **(Fig 3B)**. Additionally, lipid metabolic process, lipid transport, muscle organ development and cellular amino acid catabolic process were uniquely enriched in stage 2 biological processes.

### Blood markers for Stage 1 and Stage 2 r-HAT in Malawi

**Fig 4:**
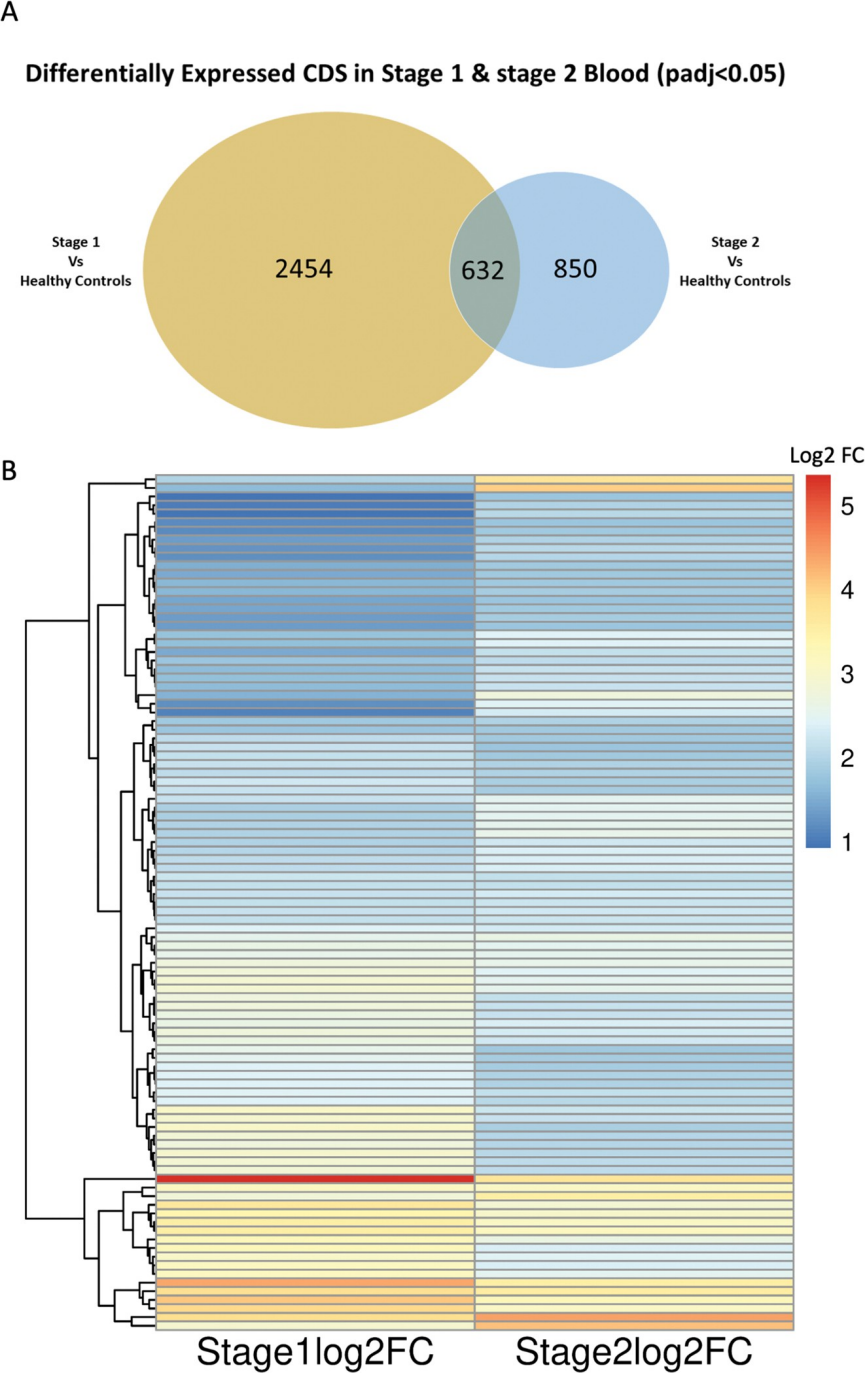
Comparison of protein coding genes differentially expressed in Stage 1 and Stage 2 blood (padj < 0.05). **(A)** Number of DE protein coding genes found in both Stage 1 and Stage 2 cases. **(B)** Hierarchical clustering heatmap of DEGs intersecting in Stage 1 and stage 2 blood. For the heatmap legend color, dark blue represents genes upregulated with log2FC 1 and red represents genes upregulated with log2FC > 5. See S1 and S2 Files for gene names and their corresponding log2 FC.

Next, we compared significantly expressed (padj<0.05) protein coding transcripts in stage 1 (2454) and stage 2 (850) blood, and we found 632 transcripts that were differentially expressed in both stages **(Fig 4A)**. *ZNF354C* was upregulated (log2FC 1.9) in stage 1 only, while in contrast, *TCN1* (log2FC 2.0) and MAGI3 (log2FC 1.4) were upregulated in stage 2 blood only **(Fig 4B)**. Besides, 71 transcripts were upregulated with log2FC >3.0 in stage 1 blood **(S4A Fig),** while *DMD*, *NOXRED1*, *HBB*, *PROK2*, *LIMS2*, *CD14* were the most upregulated (log2FC >1.9) transcripts in stage 2 blood **(S4B Fig)**. Furthermore, among other biological pathways, the DEGs were enriched for circadian rhythm and regulation of translation in stage 2 blood and translation, immune system process, viral process in stage 1 blood (**S5A and S5B Fig**).

### Neutrophils underlie differentially expressed blood cells in r-HAT disease in Malawi

**Fig 5:**
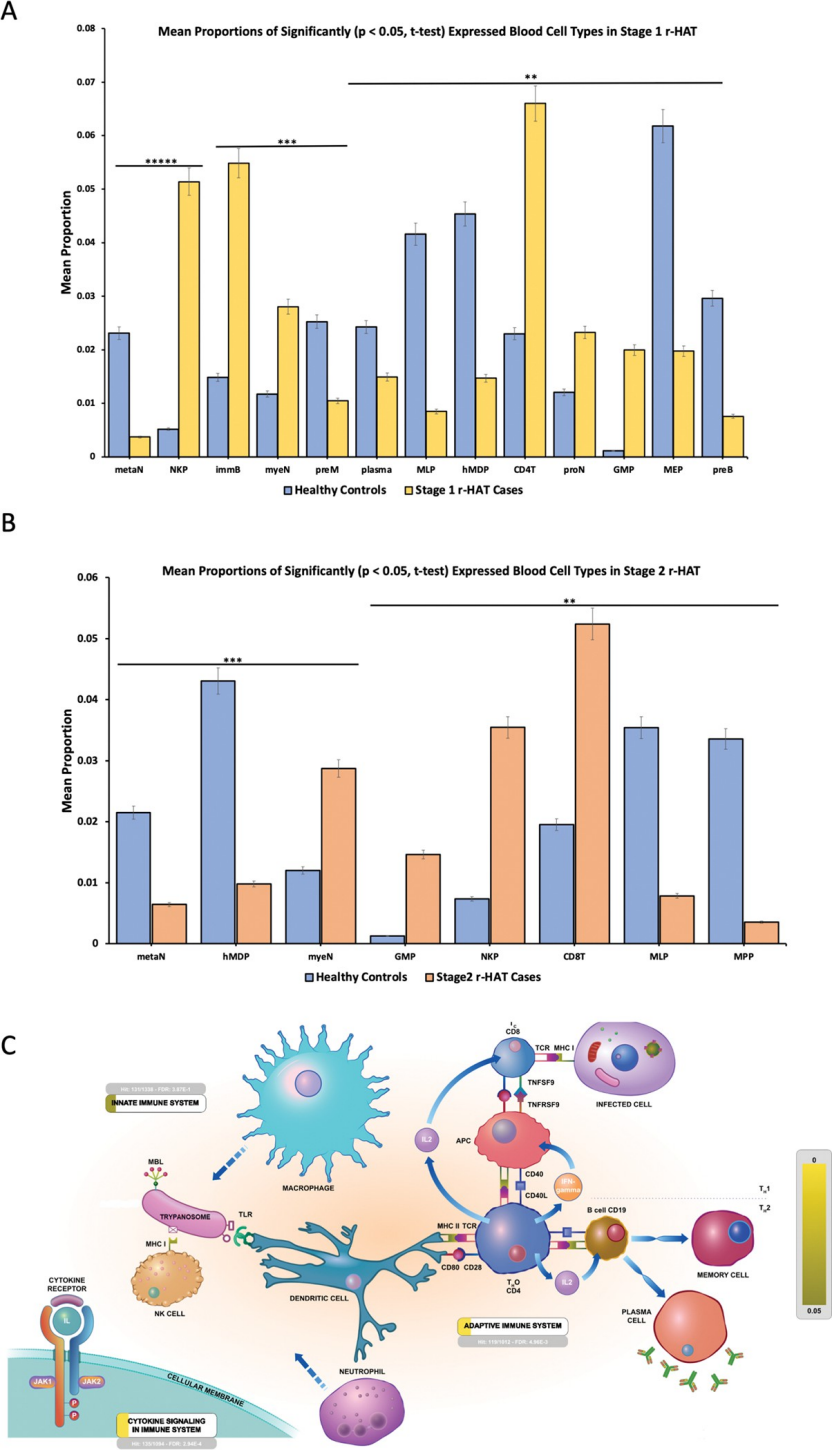
Immune system Blood cells activated in r-HAT. **(A and B)** Blood cell types that had significantly (p<0.05, t-test) different proportions in stage 1 cases versus controls and stage 2 versus controls respectively. See S3 Table for full cell type names. *****p < 3E-05, ***p < 9E-03 and **p<0.05. **(B)** Visualized output of innate and adaptive immune system pathways interaction in r-HAT cases based on DEGs loaded into Reactome pathway database (17). Yellow represents more significant and darker yellow less significant. Macrophages, neutrophils and NK cells participate in innate immune response which results in significant activation of cytokine signaling pathway (FDR: 2.94E -4). Dendritic cells link innate immune system and activation of adaptive immune system through activation of CD4+ TH cells. Activated CD4 cells release IL2 and IFN-gamma that activates CD8+ T cells and B cells to differentiate into plasma cells for antibody production.

The transcriptional map of human blood cells provides a comprehensive understanding of physiological haematopoiesis [16]. We used a custom R script that normalised read counts produced by DESeq2 to obtain the proportions of different leukocyte types present in each sample. In a principal component analysis of the data, PC1 largely separated cases from controls and explained 25% of the variance in the data (**S6A Fig**). The transformed bulk RNAseq to single cell proportions data had the expected normal distribution (**S6B Fig**). Next, Stage 1 and stage 2 cases were compared against controls **(S7A and S7B Fig)**. This identified 13 and 8 blood cell types with significantly different relative abundance (p<0.05) in stage 1 and stage 2 r-HAT cases versus controls, respectively **(Fig 5A and 5B, and S3 Table)**. Lastly, all upregulated protein coding genes in Stage 1 and Stage 2 r-HAT cases were subjected to the reactome immune system pathway visualisation [17]. This identified neutrophils and macrophages as one of the early responders to trypanosome infection as well (**Fig 5C**).

## Discussion

In this study, we have presented transcriptome profiles from peripheral blood of stage 1 and stage 2 r-HAT cases versus uninfected controls in Nkhotakota and Rumphi foci in Malawi. We also compared transcriptome profiles of r-HAT cases between Nkhotakota and Rumphi foci. Although 64 blood samples from r-HAT cases were collected for this study, RNA-seq data was obtained from blood of 23 cases and 28 heathy controls due to technical challenges in sample processing and storage after collection. Nonetheless, we have presented human blood transcriptome profiles from endemic r-HAT samples in Malawi which may add to the current understanding of rhodesiense sleeping sickness.

Our data also showed activation of the innate immune system in both stages of the disease. We also identified that cases blood were enriched with myelocytes, pro-myelocytes, natural killer progenitor, immature B lymphocyte and CD8 T-helper which are central in coordinating and effecting an innate immune response. Enrichment of neutrophil precursors in blood is indicative of acute inflammation [18], which is consistent with proinflammatory profiles in r-HAT [10]. Circulating neutrophil life span is about 48hrs, at the same time BMP6 which plays a critical role in cell proliferation and type II cytokine regulation through JAK2 signalling pathway [19], was significantly expressed and upregulated in stage 1 and stage 2. Additionally, promyelocytes and myelocytes which are second and third stage of neutrophil granulopoiesis respectively [20], had high and low proportions in cases and controls respectively; whereas, metamyelocytes which are fourth stage of granulopoiesis were high in controls and low in cases. Speculatively, this might suggest that innate immune response through neutrophil activation might have a central role in responding to blood parasitaemia in Malawi r-HAT patients. Candidate genes in neutrophil activation have also been identified to respond to *Trypanosoma congolense* infection in cattle [21]. Whereas, in mice infected with *T*. *brucei brucei* (*T*. *b*. *brucei*) neutrophils were recruited at the site of tsetse fly bite but were not able to immobilise motile trypanosomes rather aided in the establishment of *T*. *b*. *brucei* blood infection [22]. This implicates the dynamic role of neutrophils in responding to various trypanosome parasite infections in different mammalian hosts and future research should consider delineating the role of neutrophils in human *T*. *b*. *rhodesiense* infections.

*IL21* and *IFI16* were also differentially expressed and upregulated in both stages of r-HAT. *IFI16* has a critical role in the interaction between the innate immune system and cellular transcriptional regulation through pattern recognition of pathogens [23]. Whereas *IL21* function is mediated by binding to its receptor, IL21R which is expressed in various immune cells such as NK cells, macrophages, B and T cells [24]. The main source of *IL21* is T follicular helper cells which are a subset of CD4+ T cells which stimulate T-cell dependent humoral immunity, and in the current study, we observed upregulation (log2FC 1.9) of CD14+ T cell transcripts in blood from stage 2 r-HAT patients. CD14+ T cells are also involved in activation of macrophages and regulation of macrophage metabolic profiles [25], which may be consistent with our finding of *IL21* upregulation, activated lipid metabolic process, lipid transport and cellular amino acid metabolic process in stage 2 blood only (**Fig 3B**).

Our study of clinical presentation of r-HAT in the same participants enrolled in this study had identified weight loss was significantly (p<0.01) associated with stage 2 r-HAT disease [8]. Weight loss is a key characteristic of cachexia which result in depletion of lipid droplets in adipose tissue and muscle due to increased lipid metabolism coupled with reduced lipid uptake and lipogenesis [26,27]. Interestingly, blood of stage 2 cases were enriched for transcripts involved in lipid metabolic process, lipid transport and muscle organ development. Consistently, a study in horses infected with *T*. *evansi* and having cachexia were found to have increased blood levels of lipoprotein and triglyceride which are evidence of lipolysis [28]. Similar studies in rabbits that became emaciated after being infected with *T*. *b*. *brucei* also showed hypertriglyceridemia due to defective triglycerides clearance in blood circulation [29]. Cachexia in infectious disease is proposed to be effectuated by cytokines that stimulate the NF-kB and JAK-STAT signalling pathways activation (**Fig 5C**), thereby inducing various catabolic pathways in adipose tissue and muscles which favours CD4+ T cell reprogramming [30,31].

Trypanomatids also exploit folic acid in haemoglobin for folate biosynthesis thereby causing anaemia in HAT cases [32]. In this study, HBB which is required for synthesis of ß-globin and form the main structure of the human haemoglobin A, was significantly enriched in blood of stage 2 cases. Moreover, C12orf23 that encodes BRWANIN peptide which is important for respiratory chain complex III (CIII) assembly [33], was one of the most differentially expressed transcripts in both stages of r-HAT. CIII is involved in cellular redox reaction together with *b*-type cytochrome which form the haem prosthetic groups in haemoglobin, which may be consistent with upregulation of HBB in cases in the current study.

Trypanosome infections disrupt the circadian rhythm *in vivo* and *in vitro* [34], and here, we found that *CIPC* and *PER1* genes were dysregulated in stage 1 blood. This suggest that a subtle disruption of the host circadian system by *T*. *b*. *rhodesiense* may start early in infection during the hemolymphatic stage, although sleep disturbance is only observed in late stage 2 r-HAT. Although sleep disturbance is generally associated with late stage 2 r-HAT [35,36], we previously identified sleep disturbance as a significant (p<0.032) clinical sign in both stages of r-HAT in the same individuals studied here [8]. This is consistent with altered circadian rhythm in mice adipose tissue [34], suggesting that sleep disturbance seen in severe HAT disease might be due to assault of the circadian clock in both the blood and the brain [37].

Lastly, we found that *ZNF354C* was upregulated in stage 1 blood, whereas *TCN1* and *MAGI3* were only upregulated in stage 2 blood. Future research may explore whether they have a diagnostic potential of being used in combination with other blood markers to diagnose stage 1 and stage 2 r-HAT cases without need of the invasive lumber puncture collection of CSF for diagnosis of stage 2 disease. Unlike in a similar study in Ugandan r-HAT patients which identified *C1QC*, *MARCO* and *IGHD3–10* upregulated in both blood and CSF [11], these transcripts were neither upregulated nor significant differentially expressed in Malawian r-HAT patients. This supports the need for personalised medicine but not universal medicine in the treatment of r-HAT as infected individuals in different disease foci respond differently to trypanosome infection.

In conclusion, this study compared differentially expressed transcripts in blood of stage 1 and stage 2 r-HAT cases from sleeping sickness endemic foci of Malawi. We identified transcripts that were significantly differentially expressed and upregulated in each stage of the disease. We also identified neutrophil precursors having the most significant difference in blood levels from r-HAT patients with both stages of the disease, and macrophages as possible responders in blood of patients with late-stage disease. We highlighted that weight loss in r-HAT may be consistent with enrichment of lipid metabolic process transcripts associated with cachexia in infectious diseases, and we propose adoption of routine measurement of lipid metabolic profiles for early characterisation and management of cachexia in r-HAT. We have also identified transcripts that in combination with other markers might be explored in future research for staging of r-HAT in Malawi without the need of lumber puncture even considering that such staging shall not be confounded on duration of infection rather than CSF invasion. Our study has provided insights into human responses to trypanosome infection in Malawian r-HAT patients which may add to the current understanding of sleeping sickness disease. In this study, we did not investigate transcriptome profiles in CSF of stage 2 individuals, hence, future studies should consider determining the human transcriptome profiles in CSF of stage 2 cases which may provide insights into central nervous system invasion in Malawian r-HAT.

## Methods

### Ethics statement

Ethical approval of the study was obtained from the Malawi National Health Sciences Research Committee (Protocol Number: 19/03/2248). Written consent and assent (with guardian approval) were obtained from each study participant before sample collection.

### Study sites and sample collection

We have recently described r-HAT surveillance and study participants recruitment [8]. Briefly, sample collection was done during active and passive r-HAT surveillances conducted for 18 months from July 2019 to December 2020. Both r-HAT cases and healthy controls were confirmed to be infected with trypanosome parasites or not by either microscopic examination of thick blood films or microhematocrit centrifugation during the surveillance period. Additionally, a PCR to detect the SRA gene of *T*. *b*. *rhodesiense* parasites was done to confirm *T*. *b*. *rhodesiense* species in r-HAT cases or to validate trypanosome negative status of recruited controls as previously described (10). Staging of r-HAT was done by microscopic examination of CSF pellet for either trypanosome parasites or white blood cell count >5 leucocytes/μl after a single centrifugation of CSF at 6000 rpm for 10 minutes. Upon obtaining consent, 2ml whole blood samples were collected into PAXgene tubes from r-HAT cases and matching trypanosome negative healthy individuals and stored at -20°C until processing. Healthy controls were matched for sex, age group and disease foci. For r-HAT positive individuals, samples were collected before initiation of HAT treatment and all patients were thereafter treated following the national HAT treatment guidelines.

### RNA sequencing and analysis

RNA was extracted from the preserved PAXgene blood as previously described [38]. A minimum of 1μg of total RNA was shipped to the Center for Genomics Research at the University of Liverpool for sequencing. Samples were checked for quality using an Agilent Bioanalyzer and samples with RNA < 1μg were excluded. Libraries were prepared from total RNA using the QIASeq FastSelect rRNA, Globin mRNA depletion and NEBNext Ultra II Directional RNA Library Prep Kit and were sequenced to a target depth of 30 million reads on the Illumina NovaSeq (100 million reads for samples infected with *T*. *b*. *rhodesiense* parasites). FASTQ reads were aligned to the GRCh38 release 84 human genome sequence obtained from Ensembl [39] using HiSat2 [40] and annotated using the *Homo sapiens* GRCh38.104.gtf file from Ensembl. Genes that were differentially expressed between phenotypes were identified using DESeq2 [41]. The proportions of different cell types in each sample were estimated using Bisque [42]. Single cell reference sequence data from bone marrow and peripheral blood from Chinese donors was obtained from 7551 individual human blood cells representing 32 immunophenotypic cell types [16]. Network analysis of enriched genes was done using XGR, InnateDB and ExpressAnalyst [13].

## Supporting information

S1 Fig Differential gene expression in r-HAT cases vs controls. **(A)** Sample to sample hierarchical clustering heatmap with complete linkage of cases vs controls. **(B)** Radial plot of the distribution and interception of DEGs in Stage1 and Stage 2 blood vs control blood. Grey color represents genes that were not significant; red represents genes enriched in stage 1 only; green represents genes enriched in stage 2 only; blue represents genes enriched in controls only; light blue genes in cases and control; pink represents genes enriched in both stage1 and controls; and yellow represents genes enriched in both stage 1 and stage 2 blood.
(TIF)

S2 Fig Stratification of Differentially Expressed genes (DEGs) using Principal component analysis (PCA) values for r-HAT cases vs healthy controls grouped into males and females. Individuals aged <10 years were within confidence ellipse for each group.
(TIFF)

S3 Fig Significant differentially expressed genes. Gene types that were significant differentially expressed in Stage 1 **(A)** and Stage 2 **(B)** r-HAT. Protein coding genes were the most differential expressed followed by lcnRNA and pseudogenes in both stage 1 and 2 r-HAT.
(TIF)

S4 Fig List of genes specifically upregulated in blood of stage 1 (log2FC > 3.0) only **(A)**, and **(B)** in stage 2 (B, log2FC > 1.5) only.
(TIF)

S5 Fig Biological pathways enriched in genes DE in Stage 1 blood only **(A)** and in Stage 2 blood only **(B)**. Images generated by ExpressAnalyst.
(TIF)

S6 Fig Stratification of single blood cells in cases versus controls. **(A)** First and second principal components of the proportions of different cell types by phenotype. The cases and controls are separated along PC1. **(B)** Plots showing normal distribution of the transformed bulk RNA-Seq data into blood single cell RNA-seq data.
(TIF)

S7 Fig Stratification of single blood cells in Stage 1 and stage 2 r-HAT cases versus controls on PC1 and PC2. (TIF)

S1 Table Demographic details of recruited study participants. (TIF)

S2 Table PCA2GO Functional Annotation of Immune biological functions significantly enriched p<10E-10) in Stage 1 and stage 2 r-HAT cases. DEGs = Differentially expressed genes.
(TIF)

S3 Table Mean proportion and p-values (t-test) of cell type for Stage 1 cases, Stage 2 cases and controls. (TIF)

S1 File List of significant (padj <0.05) differentially expressed genes in Stage 1 r-HAT with their corresponding log2 fold change. (XLSX)

S2 File List of significant (padj <0.05) differentially expressed genes in Stage 2 r-HAT with their corresponding log2 fold change. (XLSX)
