# Supplementary figures and images for: Differences in gene expression profiles in early and late stage rhodesiense HAT individuals in Malawi
Source: PLoS Negl Trop Dis. 2023 Dec 6;17(12):e0011803. doi: 10.1371/journal.pntd.0011803 (PMC10727365; doi:10.1371/journal.pntd.0011803)

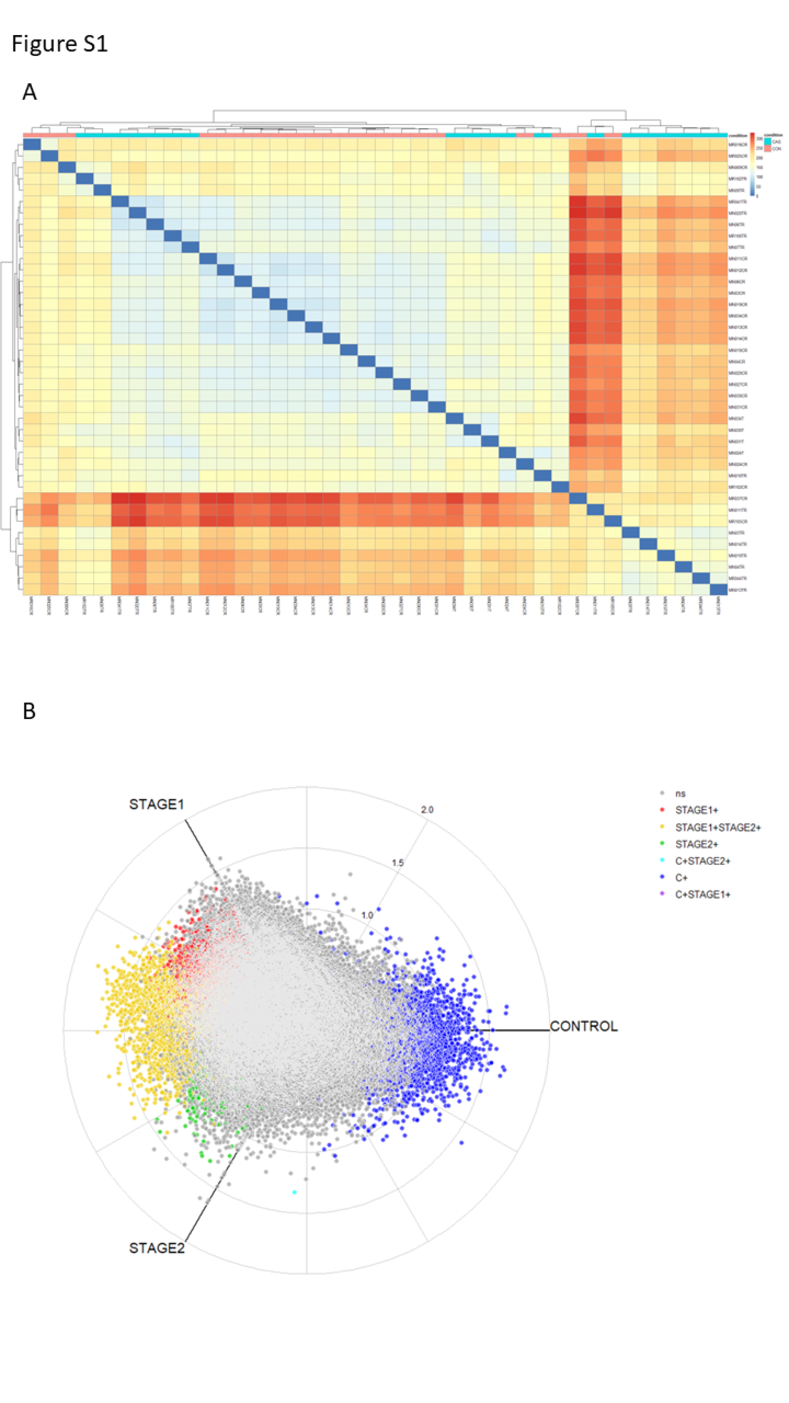

Supplement: S1 Fig — (A) Sample to sample hierarchical clustering heatmap with complete linkage of cases vs controls. (B) Radial plot of the distribution and interception of DEGs in Stage1 and Stage 2 blood vs control blood. Grey color represents genes that were not significant; red represents genes enriched in stage 1 only; green represents genes enriched in stage 2 only; blue represents genes enriched in controls only; light blue genes in cases and control; pink represents genes enriched in both stage1 and controls; and yellow represents genes enriched in both stage 1 and stage 2 blood. (TIF) [file pntd.0011803.s001.tif]

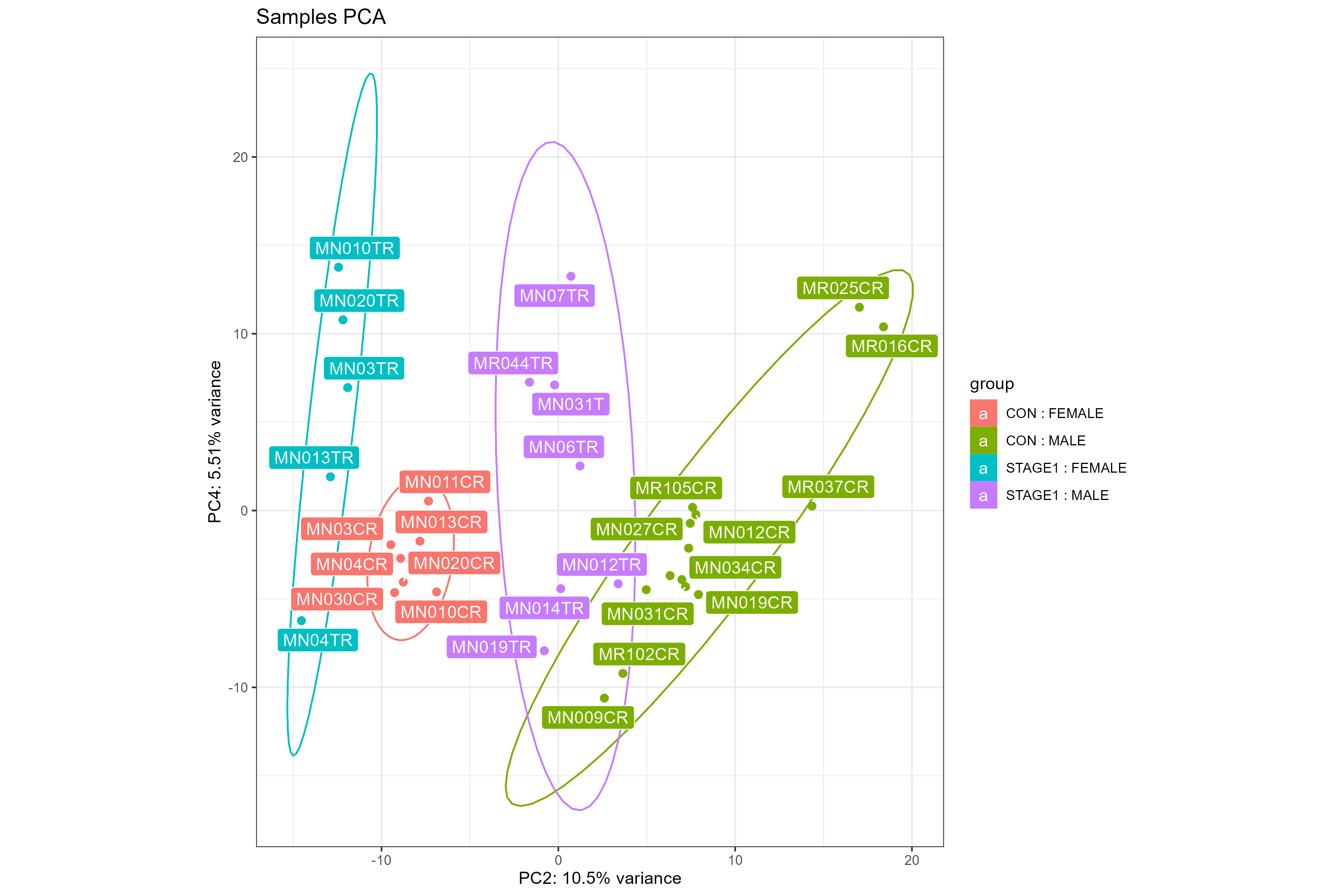

Supplement: S2 Fig — Individuals aged <10 years were within confidence ellipse for each group. (TIFF) [file pntd.0011803.s002.TIFF]

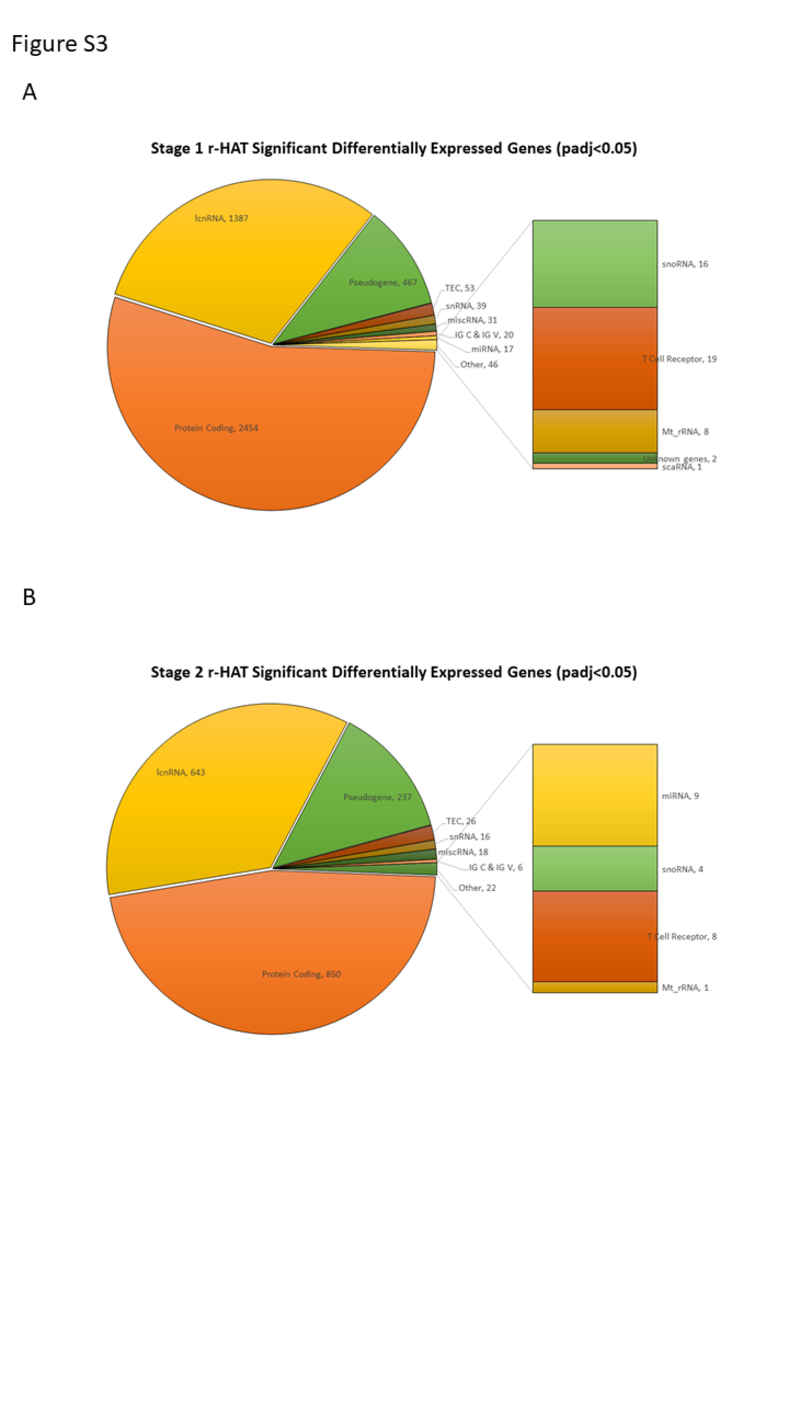

Supplement: S3 Fig — Gene types that were significant differentially expressed in Stage 1 (A) and Stage 2 (B) r-HAT. Protein coding genes were the most differential expressed followed by lcnRNA and pseudogenes in both stage 1 and 2 r-HAT. (TIF) [file pntd.0011803.s003.tif]

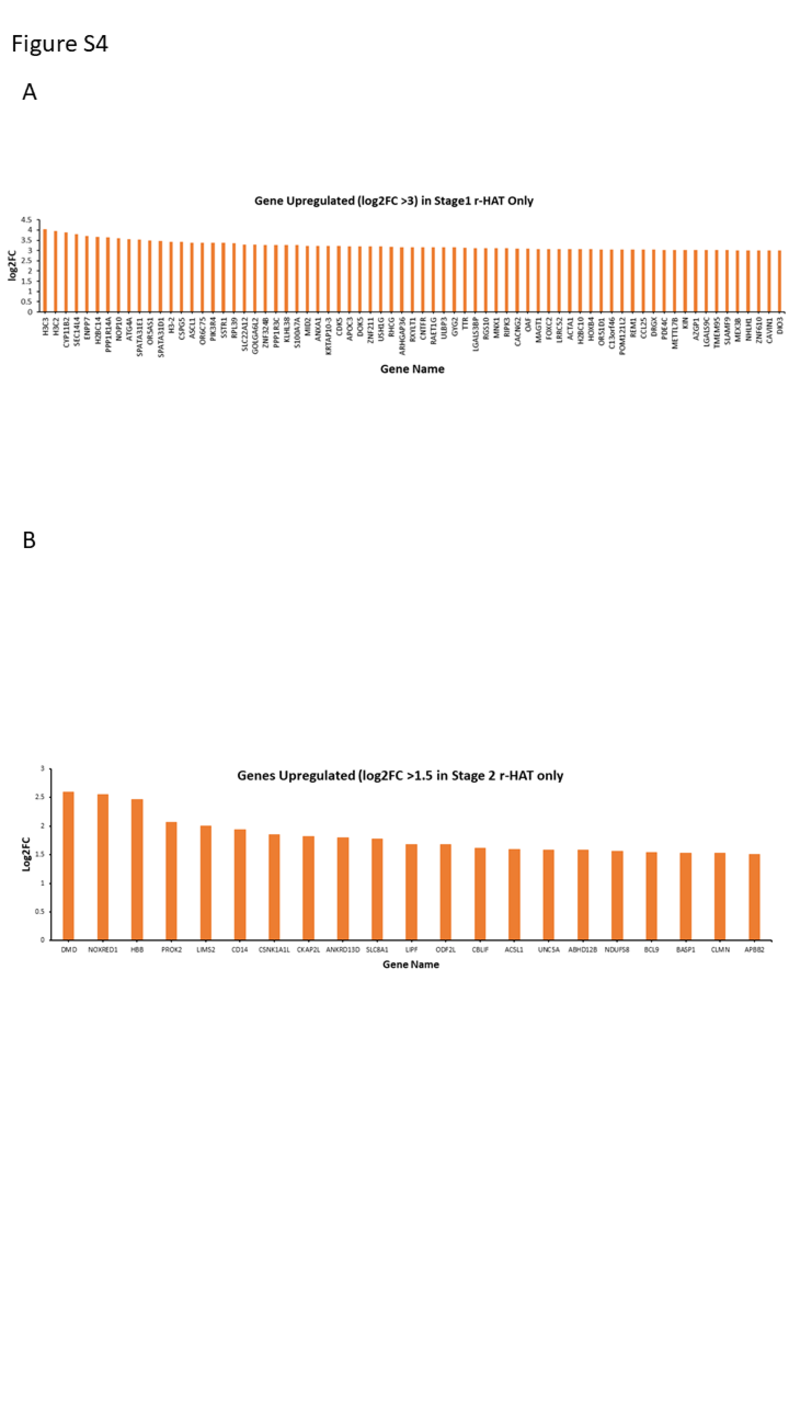

Supplement: S4 Fig — List of genes specifically upregulated in blood of stage 1 (log2FC > 3.0) only (A), and (B) in stage 2 (B, log2FC > 1.5) only. (TIF) [file pntd.0011803.s004.tif]

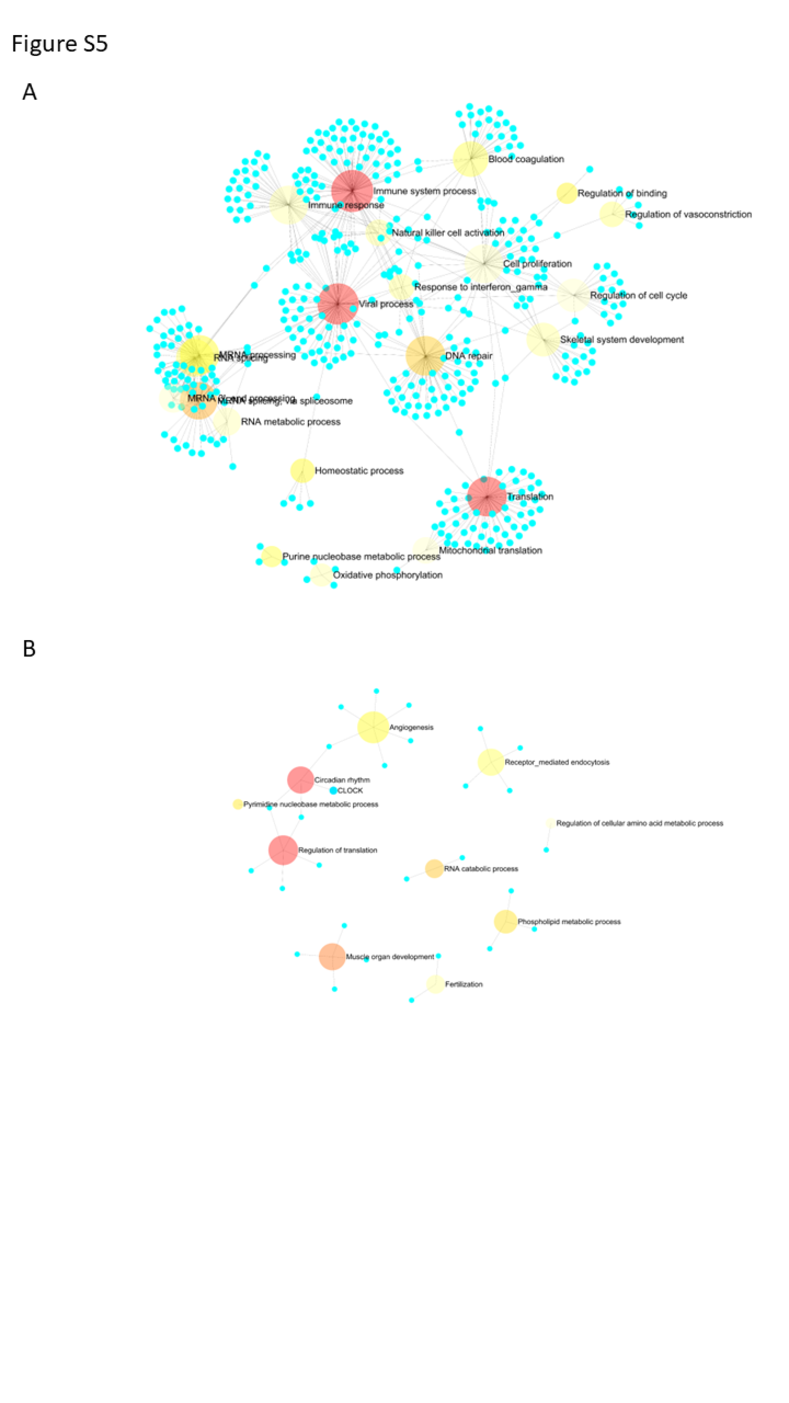

Supplement: S5 Fig — Biological pathways enriched in genes DE in Stage 1 blood only (A) and in Stage 2 blood only (B). Images generated by ExpressAnalyst. (TIF) [file pntd.0011803.s005.tif]

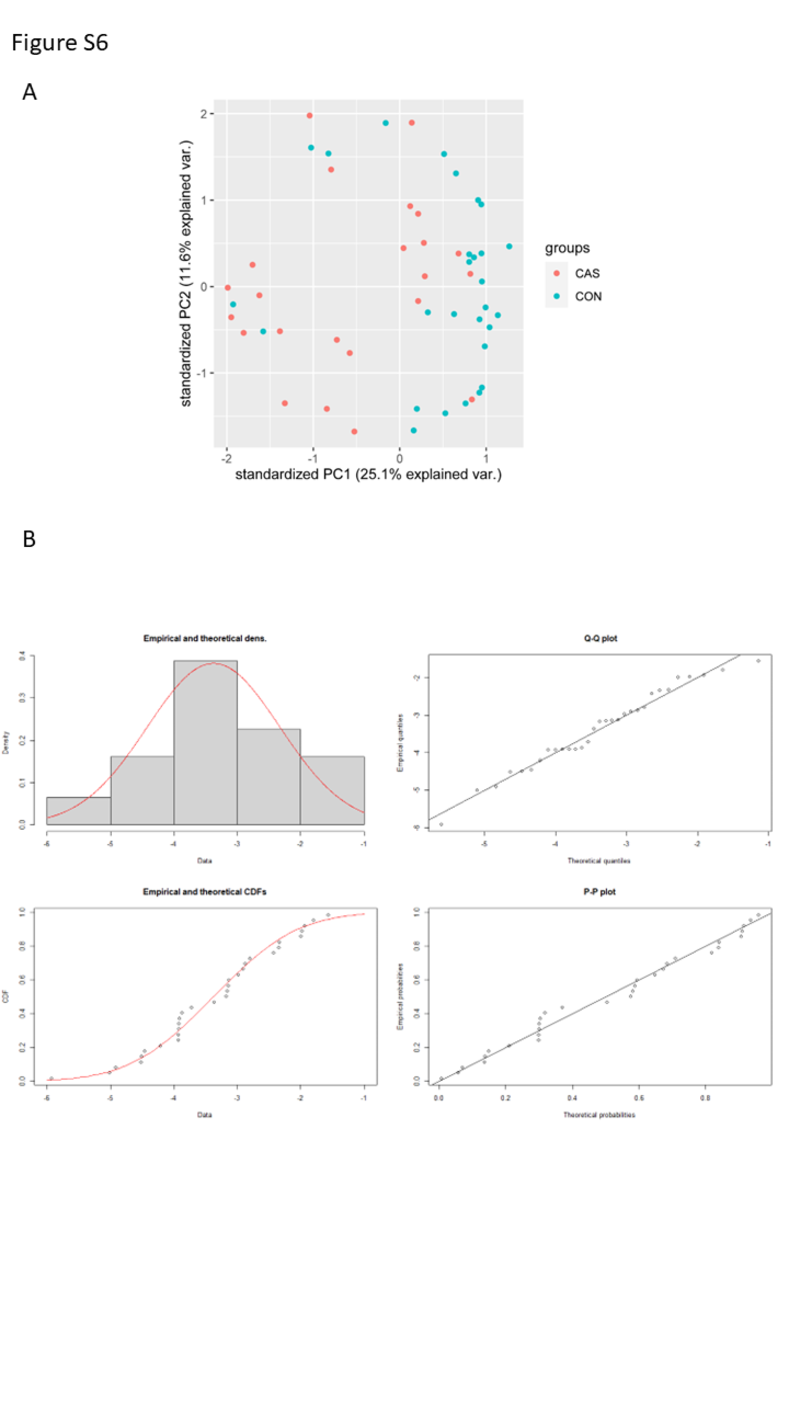

Supplement: S6 Fig — (A) First and second principal components of the proportions of different cell types by phenotype. The cases and controls are separated along PC1. (B) Plots showing normal distribution of the transformed bulk RNA-Seq data into blood single cell RNA-seq data. (TIF) [file pntd.0011803.s006.tif]

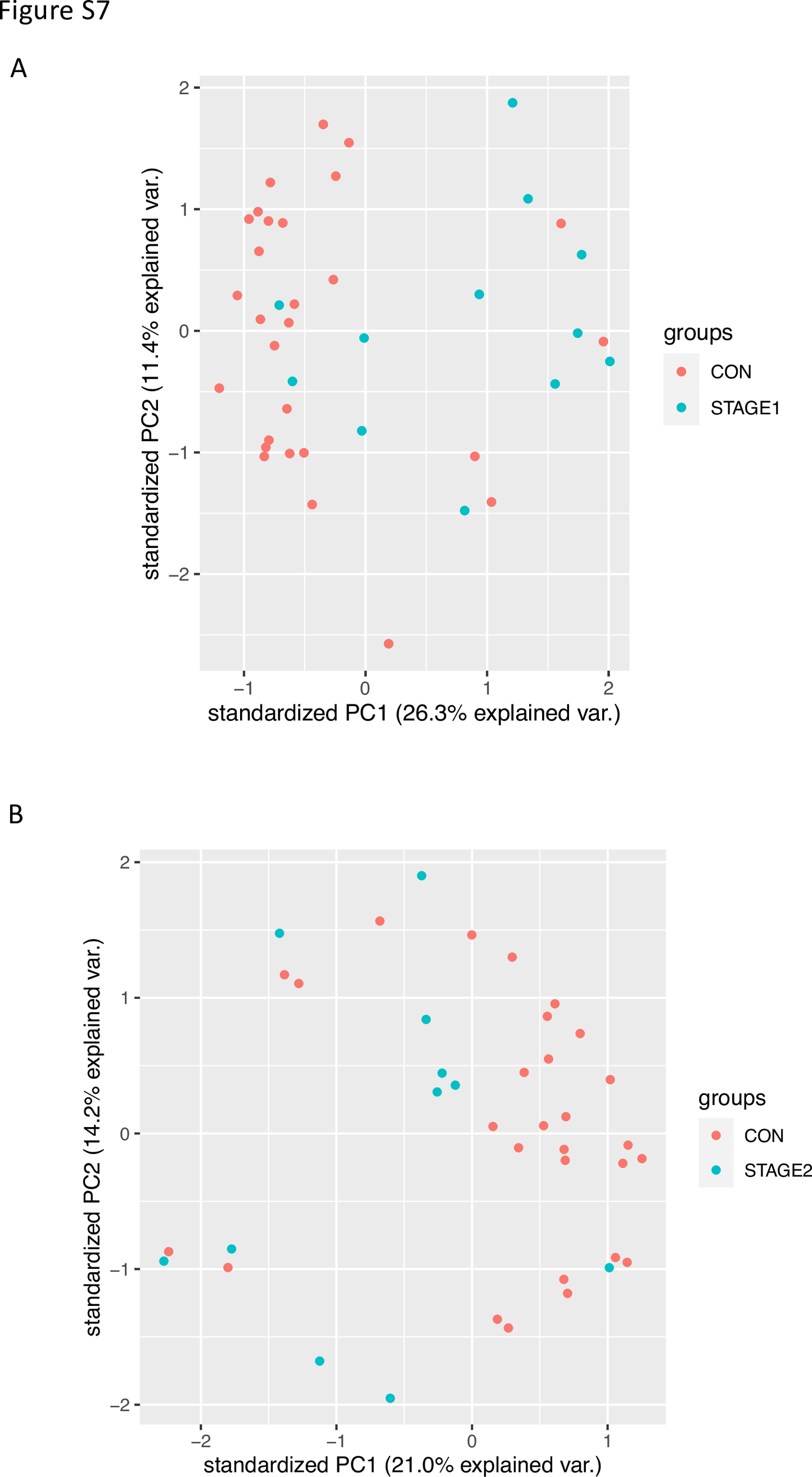

Supplement: S7 Fig — (TIF) [file pntd.0011803.s007.tif]

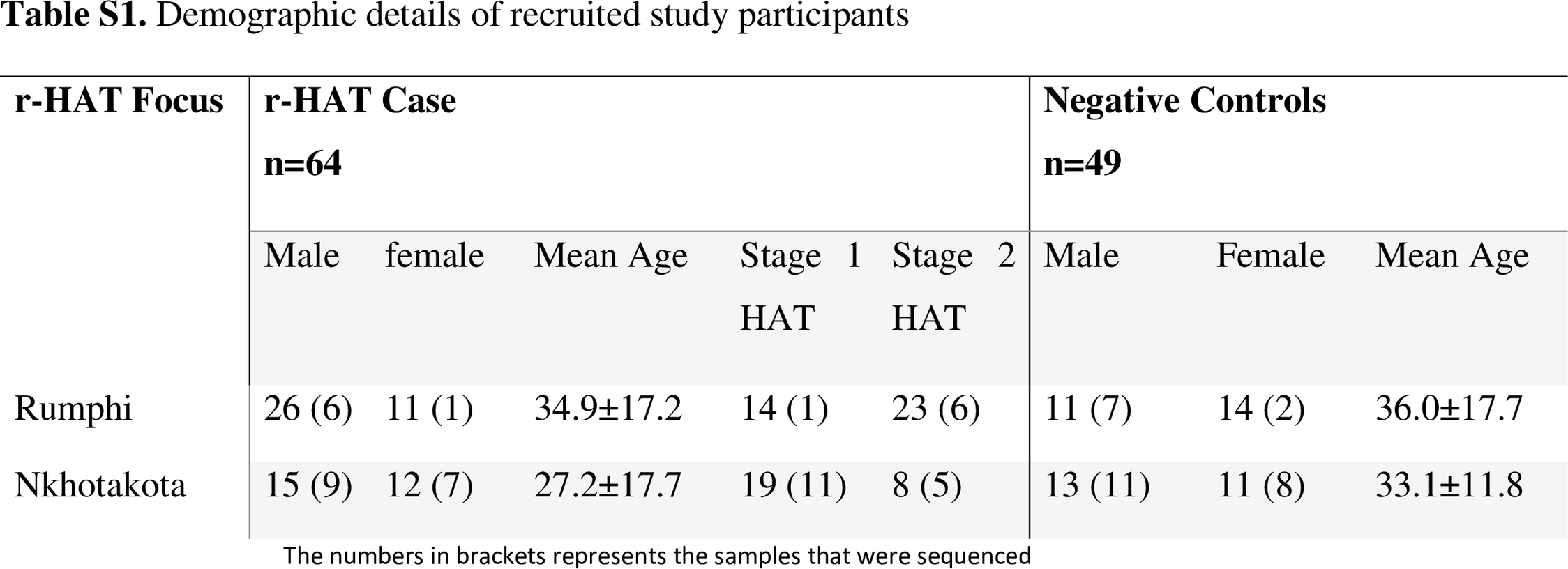

Supplement: S1 Table — (TIF) [file pntd.0011803.s008.tif]

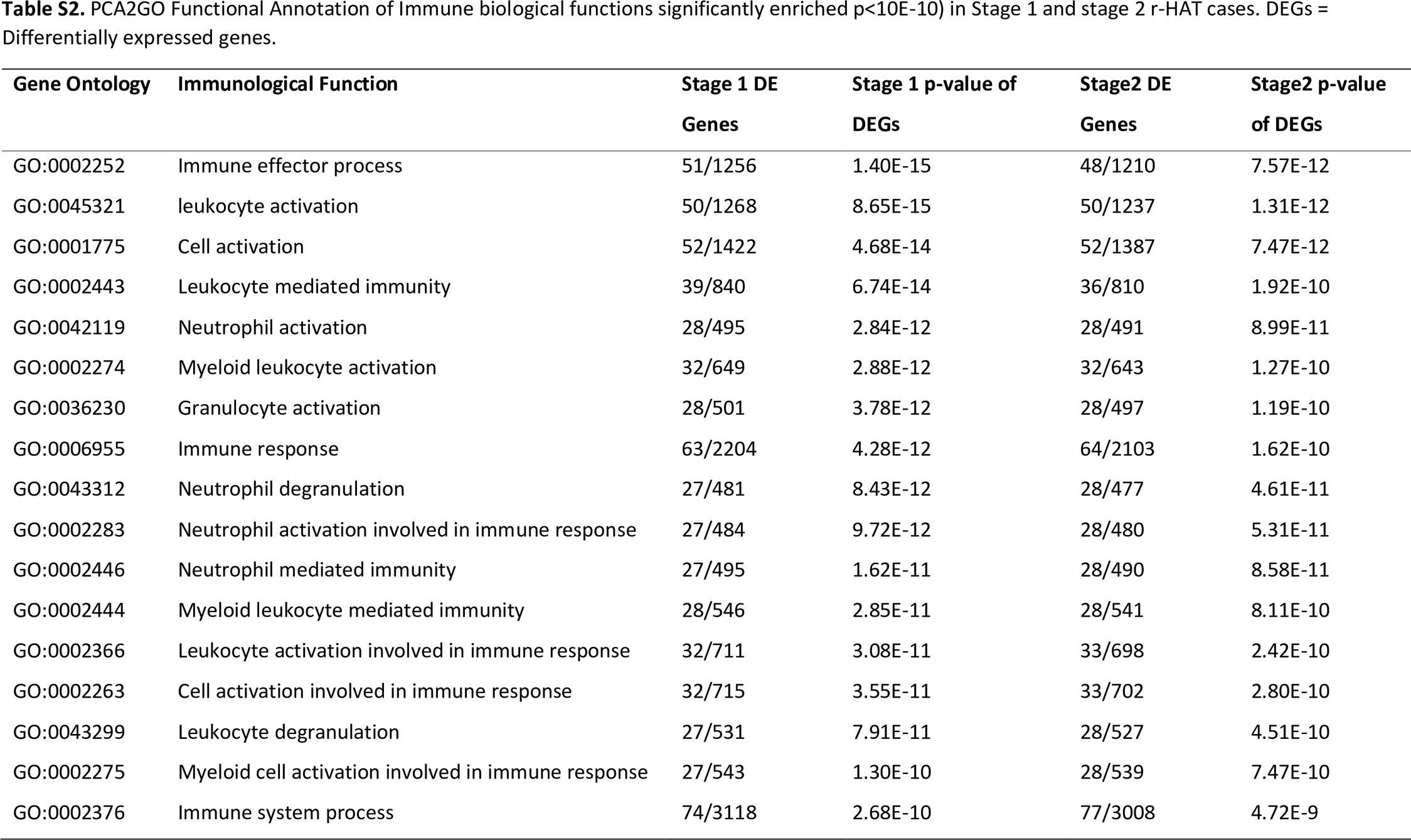

Supplement: S2 Table — DEGs = Differentially expressed genes. (TIF) [file pntd.0011803.s009.tif]

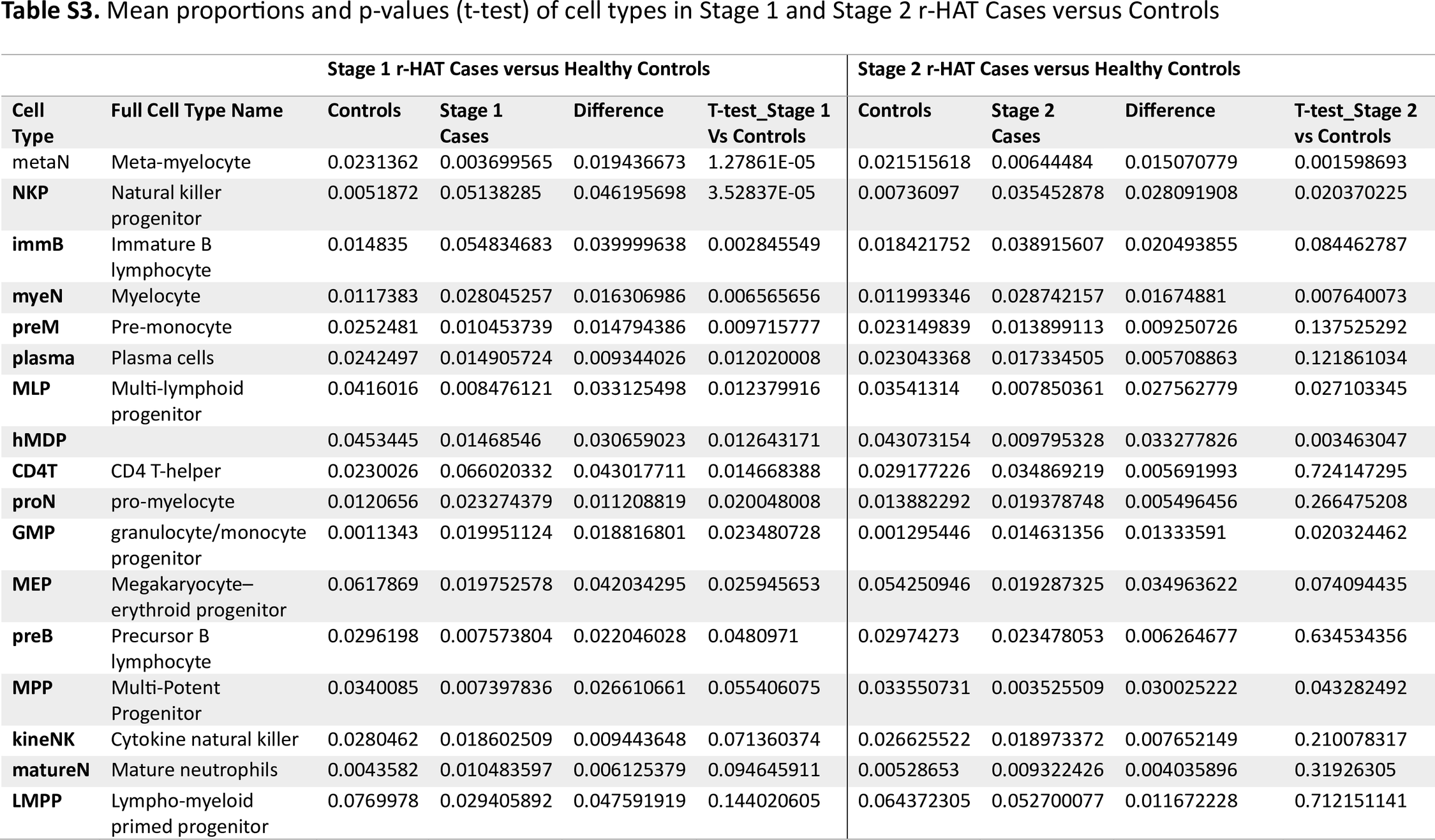

Supplement: S3 Table — (TIF) [file pntd.0011803.s010.tif]
